# Aluminum Matrix Composites Manufactured using Nitridation-Induced Self-Forming Process

**DOI:** 10.1038/s41598-019-56802-3

**Published:** 2019-12-31

**Authors:** Kon-Bae Lee, Sung-Hoon Kim, Dae-Young Kim, Pil-Ryung Cha, Hae-Sung Kim, Hyun-Joo Choi, Jae-Pyong Ahn

**Affiliations:** 1 School of Advanced Materials Engineering, Kookmin University, 02707 Seoul, South Korea; 2 Advanced Analysis Center, Korea Institute of Science and Technology (KIST), 02792 Seoul, South Korea; 3 SENUS Co., Ltd, 14447 Bucheon, South Korea

**Keywords:** Engineering, Materials science

## Abstract

Conventional manufacturing processes for aluminum matrix composites (AMCs) involve complex procedures that require unique equipment and skills at each stage. This increases the process costs and limits the scope of potential applications. In this study, a simple and facile route for AMC manufacturing is developed, a mixture of Al powder and the ceramic reinforcement is simply heated under nitrogen atmosphere to produce the composite. During heating under nitrogen atmosphere, the surface modification of both Al and the reinforcement is induced by nitridation. When the oxide layer covering Al powder surface is transformed to nitrides, temperature in the local region increases rapidly, resulting in a partial melt of Al powder. The molten Al infiltrates into the empty space among Al powder and reinforcement, thereby enabling consolidation of powders without external forces. It is possible to fabricate AMCs with various types, sizes, volume fractions, and morphologies of the reinforcement. Furthermore, the manufacturing temperature can be lowered below the melting point of Al (or the solidus temperature for alloys) because of the exothermic nature of the nitridation, which prevents formation of un-wanted reactants. The relative simplicity of this process will not only provide sufficient price competitiveness for the final products but also contribute to the expansion of the application scope of AMCs.

## Introduction

Since the development of the first modern composite material, fiberglass, composite materials with excellent and/or a unique combination of properties that are not attainable from traditional monolithic materials (e.g., metals, ceramics, and polymers), are now recognized as essential materials for various applications^1–4^. One of them is metal matrix composites (MMCs), and among the various metals, aluminum (Al) matrix composites (AMCs) account for about 70% of the current commercial MMCs market^5–7^. Through decades of continuous research and development, considerable technological advances have been made in the manufacturing of AMCs, which are now being used not only by high-tech industries, such as land transportation (automotive and railway), thermal management, aerospace, industrial, recreation and infrastructure, but also in our daily life^1–10^.

Because AMCs are not solid solutions like metal alloys, one of the most important requirements in their production is to improve the wettability between Al and the reinforcement. However, because wettability is an intrinsic property of materials, and generally, the wettability between Al and a ceramic reinforcement is poor, it is not an overstatement that the history of AMC development has been developed by various attempts to overcome the poor wetting. Therefore, efforts have been made to achieve this via various strategies including high-energy stir casting (i.e., casting)^11–19^, infiltrating molten Al into the ceramic preform under high pressure (infiltration method)^20–26^, and consolidating a mixture of Al and reinforcement powders (powder metallurgy)^27–32^, and formation of reinforcement in the Al matrix via *in-situ* reaction (i.e., nitridation)^33–36^. These processes account for almost 90% of the production techniques adopted in the global market for manufacturing AMCs^6,7^. However, these methods are essentially involve several stages in the manufacturing process and require additional equipment or catalysts to perform each stage^22–25^. These are key factors in reducing the competitiveness of AMC products owing to the increase in the manufacturing costs. Moreover, the major commercial techniques have limitations on the type and volume fraction of reinforcement that can be applied owing to the nature of the process. Furthermore, each additional process step can have a detrimental effect on the properties of the final product^1–10^.

As mentioned earlier, the utmost advantage of composite materials, including AMCs, is that various combinations of the matrix and reinforcement can be exploited to achieve excellent properties that are not obtainable with monolithic materials and can be tailored to the desired properties. Despite these advantages, the AMC market is very small compared to that of its competitors (e.g., Al alloys) primarily owing to its poor price competitiveness^1–10^. Therefore, if a new, price-competitive manufacturing process is developed, then the application fields and markets for AMCs will be further expanded.

In this study, we introduce a new and very simple yet innovative process that refutes the conventional AMC manufacturing. The poor wettability between the matrix and reinforcement in AMCs manufacturing is a challenge to be solved, but so far, all of the research efforts have attempted to solve the wettability-associated problems by external means (e.g., application of pressure). In this study, however, we have developed a simple AMC production method based on a new concept that utilizes an exothermic reaction accompanied by nitridation without using external mechanical means, pressure, or catalyst, which are used in conventional processes^37^. Here, the AMCs can be manufactured by a simple process, which drastically reduces the complex multi-step manufacturing process that cannot be avoided in conventional commercial processes for manufacturing AMCs. Specifically, when Al powder and any desired reinforcement are mixed and heated under nitrogen atmosphere, AMCs are fabricated irrespective of the degree of wettability between the two phases. This is a very novel concept that has not been known or attempted so far. This simple and easy method can provide sufficient price competitiveness for the final product and may potentially contribute to expanding the applications of AMCs. We term this process “nitridation-induced self-formed aluminum composites (NISFAC) process”.

## Results and Discussion

**Figure 1:**
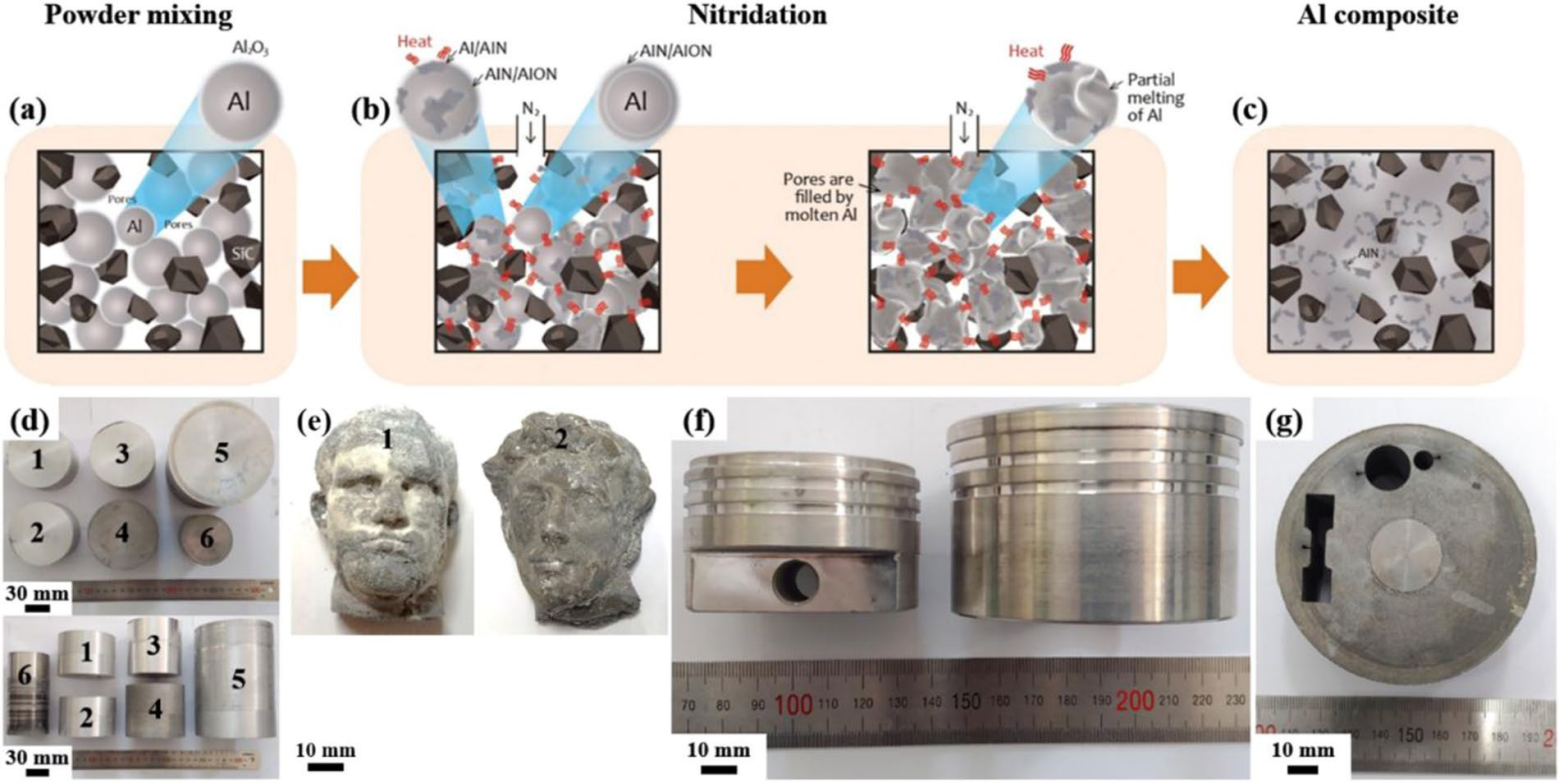
Schematics and product images of the nitridation-induced self-formed aluminum composites (NISFAC) process. **(a)** Mixing of the Al and reinforcement powders, **(b)** heating the mixed powders under a nitrogen atmosphere, **(c)** self-formed aluminum composites after heating, **(d)** unlimited product size, **(e)** net or near-net shape products (1. Agrippa, 2. Julien), **(f,g)** excellent formability.

Figure 1 describes the NISFAC process developed in this study. In contrast to the conventional processes involving multiple stages, in the NISFAC process, the AMCs are produced in one step, even when there is poor wettability between the reinforcement and matrix. During heating of the mixture of the Al powder and reinforcement under a nitrogen atmosphere, nitridation of Al occurs, as shown in Fig. 1a–c. The NISFAC process has all the advantages of powder processing. Moreover, this process has benefits in terms of up-scaling than powder processing because it does not require external forces throughout the whole process; the product size is mainly limited by the size of furnace (Fig. 1(d)) and it is easy to manufacture a net or near-net shape (Fig. 1(e)). In particular, the composites produced by the NISFAC process have excellent formability (Fig. 1(f,g)). The advantage of the NISFAC process is that the wettability between the two materials improves via a spontaneous surface modification caused by the nitridation while the Al and reinforcement are in direct contact with each other in the nitrogen atmosphere. Therefore, it is possible to easily incorporate any type of reinforcement, even in a high fraction (50% or more), to achieve the characteristics required by an application

**Figure 2:**
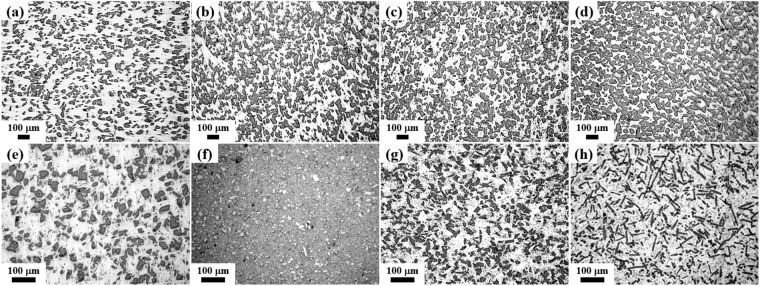
Optical images of the Al composites with various dispersed reinforcements: **(a)** 20 vol.% SiC/Al, **(b)** 30 vol.% SiC/Al, **(c)** 40 vol.% SiC/Al, **(d)** 50 vol.% SiC/Al, **(e)** 20 vol.% B_4_C/6092Al, **(f)** 10 vol.% TiB_2_/Al, **(g)** 20 vol.% Al_2_O_3_/6061Al, **(h)** 15 vol.% CF/6063Al.

Another key advantage of the NISFAC process is the ability to freely control the distribution of the reinforcement as in the case of powder metallurgy processes. Figure 2 shows the microstructure of the composites prepared by adding various types of reinforcements to pure Al and 6xxx Al alloy. As the dispersion of the reinforcement is determined at the mixing stage, if the conditions for achieving uniform dispersion are established using an appropriate mixing method, then it is possible to produce composites with a very uniform distribution of the reinforcement, as shown in Fig. 2. Thus, the degradation of the mechanical and thermal properties of AMCs due to the poor dispersion of the reinforcement is no longer a problem. In addition, various reinforcements with several unique characteristics can be added simultaneously. Therefore, by the NISFAC process, it is possible to design and manufacture many exclusive hybrid AMCs with the desired properties, similar to those obtained by alloy design (where various elements are added to achieve unique characteristics). Hence, as shown in Figs. 1 and 2, the new and economical technology provided by the NISFAC process will contribute to enhancing the applications of AMCs by overcoming the conventional process drawbacks, including complex steps, low price compatibility, limitations on the reinforcement type and volume fractions, and non-uniform reinforcement distribution.

**Figure 3:**
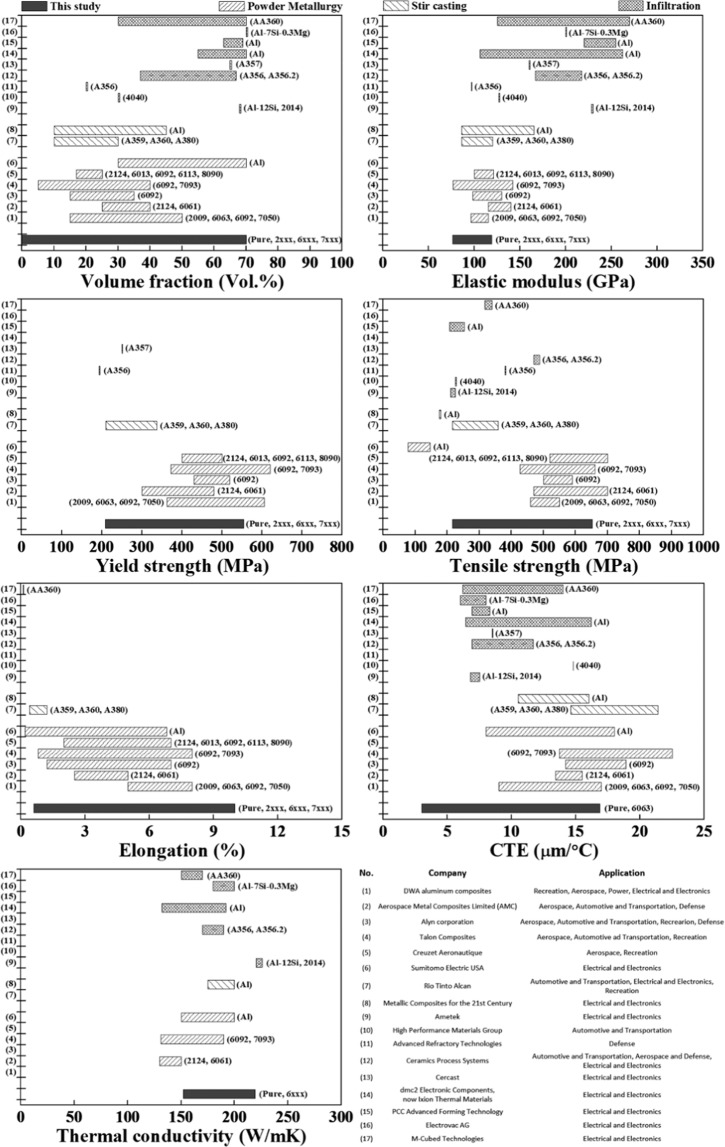
Mechanical and thermal properties of the Al/SiC_p_ composites produced in this study compared to those of other commercial Al/SiC_p_ composites.

The mechanical and thermal properties of these easily fabricated AMCs are shown in Fig. 3, and the characteristics of the Al/SiC_p_ composites prepared by typical commercial processes such as infiltration, stir casting, or the P/M method are also shown for comparison^7,9,10^. Details on the data in Fig. 3 are provided in the Supplementary information. For the commercial processes, the actual applications of the products are also listed. Although a direct comparison is difficult owing to the differences in size, volume fraction of the SiC particles, composition of the Al matrix, and limited data disclosure on the properties of the products, we can approximately compare the characteristics of the NISFAC-synthesized AMCs with the commercially produced ones. For example, the tensile properties of our samples are similar to those of the AMCs produced by the P/M method (which are evaluated to have excellent mechanical properties in comparison with those produced by other existing commercial processes). In particular, the CTE (coefficient of thermal expansion) of the NISFAC sample is very small compared to those of the conventionally processed AMCs. The detailed characteristics will be reported in another paper. This indicates that by the relatively easy, fast, and economical NISFAC process, AMCs with properties comparable to those of commercial products can be obtained. Moreover, the commercial products (used here for comparison) have been developed for specific applications and their product completeness is expected to be high. However, this is the first report of NISFACs, and a composite design is far from being established. In this context, the properties demonstrated by the AMCs synthesized by the NISFAC process can be evaluated as having sufficient competitiveness. In addition, it is expected that the characteristics of the NISFACs can be increased by further optimizing the process conditions. Summarizing the results thus far, unlike metal alloys, AMCs are not solid solutions; therefore, there is no need to consider a solubility. There is no limitation on the types of AMCs that can be manufactured, and theoretically numerous combinations (size, type, and volume fraction of both the matrix and reinforcement) are possible. However, when AMCs are manufactured using the conventional processes, the problem of wettability cannot be avoided, and it is impossible to overcome it. Contrastingly, wettability is not a concern in the NISFAC process; therefore, the composites can be manufactured in numerous combinations. This is a groundbreaking result that we can easily manufacture composites with the desired properties using various combinations. In addition, because of the simple steps involved in the NISFAC process, the equipment required for manufacturing AMCs is only a simple furnace with nitrogen atmosphere. Therefore, the NISFAC process is simple and economical and can be easily adopted without the need for unique equipment or skills.

**Figure 4:**
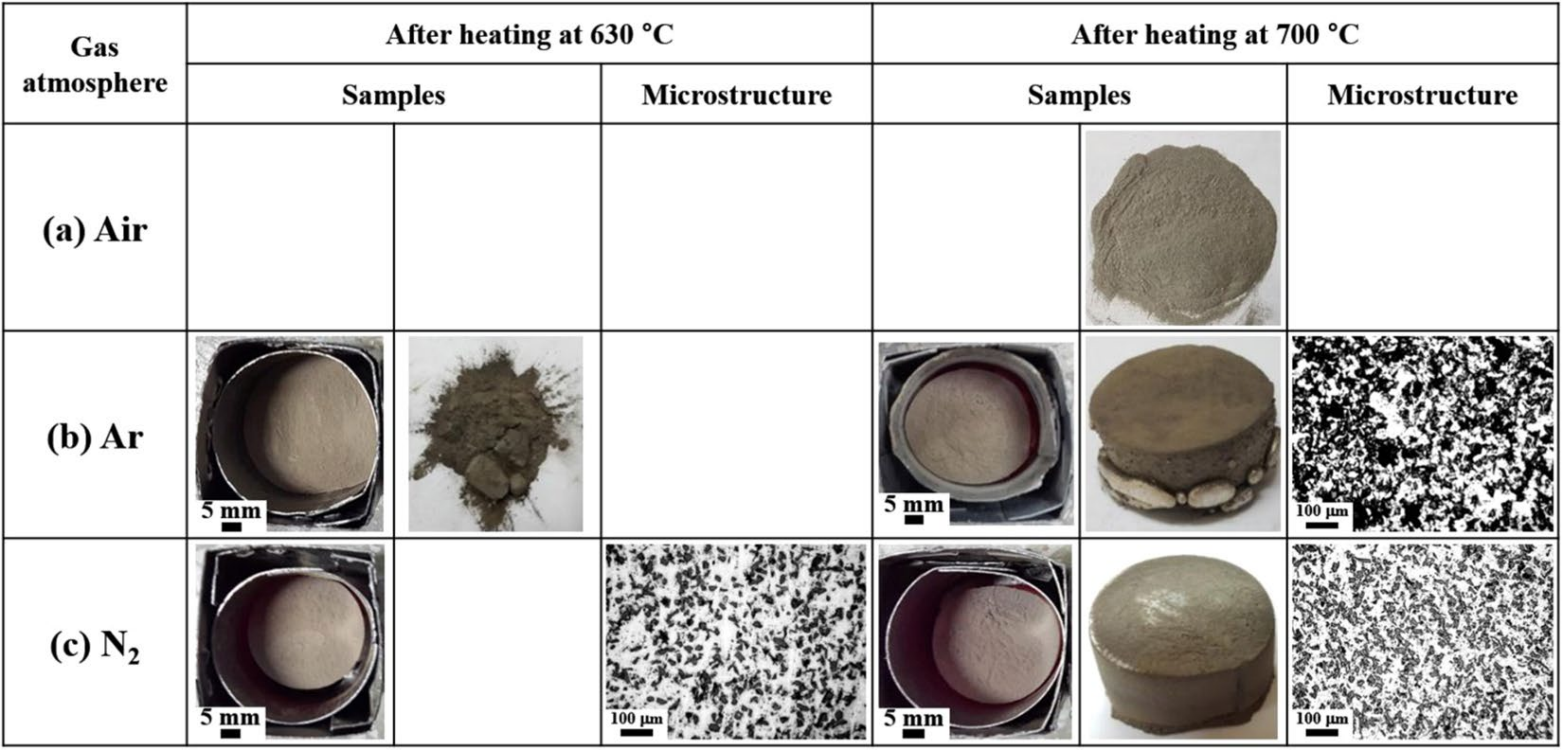
Sample images and microstructure of the Al/20 vol.% SiC_P_ composites produced at 630 °C and 700 °C in **(a)** air, **(b)** argon gas, and **(c)** nitrogen gas.

The NISFAC process began with the motivation that “AMCs could easily be made if the wettability between the two materials near the Al melting point can be increased while heating the mixed powder of Al and the reinforcement.” To demonstrate this, Al (D50: 10.37 μm) and SiC (D50: 19.8 μm, 20 vol.%) powders were mixed, placed in a graphite crucible, and heated at temperatures lower (i.e., 630 °C) or higher (i.e., 700 °C) than the melting point of Al (660 °C) for 1 h in air, argon (Ar), and nitrogen atmospheres. When heated at 700 °C in air, the Al powder retains the powder state because the molten Al phase is trapped by the surface oxide films (Fig. 4(a)). When heated in Ar atmosphere, Al melts, but a significant amount of liquid Al flows through the side of the powder bed (Fig. 4(b)). The oxide film formed on the surface of the Al powder is not sufficiently thick to suppress the ejection of the molten Al, and the liquid phase penetrates the film. As known, owing to the poor wettability between the liquid-phase Al and SiC particles^38,39^, the Al pool does not maintain its contact with the surface of the SiC particles, and hence, flows out of the bed. Contrastingly, when heated in nitrogen atmosphere, unlike the previous two cases, a composite material is produced without molten Al flowing out of the bed (Fig. 4(c)). This implies that the problem of wettability is solved by itself simply by heating the mixture of Al and SiC particles in a nitrogen atmosphere without using any external method (e.g., use of a catalyst or pressurization). During heating in N_2_, the oxide layer covering Al powder can be transformed into AlN; as calculated in Supplementary Information Fig. S1 on the basis of NIST-JANAF tables^40^, AlN phase is more stable than Al_2_O_3_ phase at the temperature in the powder bed. First, amorphous alumina layer on the surface of Al powder is transformed to γ-alumina. However, newly formed γ-alumina crystallites phases do not form a continuous layer on the surface of Al powder because of the difference in density between pure Al and amorphous alumina^41^. As a result, metallic Al will be exposed to a nitrogen atmosphere and, if the oxygen concentration is not high, healing will not occur by reoxidation. Therefore, the exposed metallic Al will react with nitrogen. As the temperature of the powder bed increases above 600 °C, significant exothermic reaction of nitridation may greatly rise a local temperature of the surface of Al powder, Al under the alumina shell may melt. The formation of liquid Al involves expansion of volume of Al powder, which accelerates destruction of aluminum shell^42,43^. The difference of coefficients of thermal expansion between Al and AlN also generates thermal stresses, which also accelerates destruction of aluminum shell^40,44,45^. The liquid Al leaks out from the AlN shell and fills the empty space of the powder bed. These results are very important in that it is possible to manufacture AMCs having the required properties by adding various reinforcement, similar to alloy design.

Interesting results are observed on heating at 630 °C (which is lower than the melting point of Al) in Ar atmosphere. The Al powder is not molten, unlike the case at 700 °C. The upper part of the powder bed is in a powdery state, and a sintered body is formed with weak bonding between the powders in the lower part (Fig. 4(b)). As opposed to the Ar atmosphere, in a nitrogen atmosphere, Al melts at a temperature even below the melting point (or liquidus line for Al alloys), resulting in composites with a relatively large shrinkage (Fig. 4(c)). These results suggest that the AMCs can be manufactured at a reasonably low temperature (even below the melting point of Al) under nitrogen atmosphere. This low production temperature is unique to this newly developed process and cannot be realized by any known commercial processes involving the melting of Al. The reduction in the manufacturing temperature of the AMCs will contribute to a reduced energy consumption and process time with subsequent reduction in the process cost. In addition, a low production temperature will improve the properties of the final composite by inhibiting or significantly reducing the formation of undesirable reaction products.

**Figure 5:**
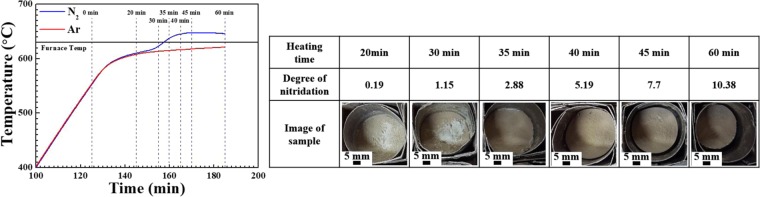
Temperature variation of the Al/SiC_p_ composites during heating at 630 °C in argon and nitrogen gas; the degree of nitridation and sample images during holding at 630 °C with time.

In the NISFAC process, AMCs are produced only in the nitrogen atmosphere (as demonstrated in our previous reports^46^), and not in the air or Ar atmospheres, because of the occurrence of nitridation during heating. To understand this, the temperature changes inside the powder bed were monitored during heating under various conditions. The typical temperature changes obtained by heating at 630 °C for 1 h separately in Ar and nitrogen atmospheres are shown in Fig. 5. The temperatures of the two beds rise nearly to 600 °C (in approximately 10 min after reaching the set temperature (i.e., 650 °C)). However, thereafter, the bed temperature in the nitrogen atmosphere rises more rapidly owing to the exothermal contribution of the nitridation, and the temperature difference between the two beds increases with the heating time. In the Ar atmosphere, because nitridation does not occur, the bed temperature continuously increases with the increase in the furnace temperature without an exothermic effect. During the 1-h hold in the Ar atmosphere, the maximum temperature inside the bed reaches approximately 621 °C, which is lower than the furnace temperature (630 °C) and melting point of Al. Thus, as shown in Fig. 4(b), the Al powder remains in the powdery state without melting. However, in the nitrogen atmosphere, the internal temperature of the bed rises owing to the exothermic nitrification, and then starts to decrease after reaching the maximum temperature of approximately 648 °C. Although the highest temperature attained is still lower than the melting point of Al, melting occurs to produce a composite. Because the temperature recorded by a thermocouple (approximately 648 °C) is macroscopic and influenced by the bulk of the sample, the actual exothermic temperature at certain points on the Al particle surfaces (where the nitridation proceeds) near the reinforcement is expected to be much higher than 648 °C. In fact, the exothermic temperature due to the nitridation varies significantly depending on the composition of the powder bed (Al particle size, type, size and volume fraction of reinforcement). Although the overall temperature was increased to 648 °C, a local temperature rise might be much more significant. In some cases, the local temperature reaches 1700 °C or higher, and thus, the stainless-steel sheath of the thermocouple melts^46–48^. Nitridation has sometimes been used to fabricate Al/AlN composites by *in-situ* reaction^37–39,46,47^. However, this new process departs from the previous work because the processing temperature is much below those suggested in the previous work ( > 1200 °C). This work intended to form AlN with small amount and small sizes. Herein, AlN was not primarily used to be reinforcement for the composites but was used to provide a local heat via exothermic reaction to locally melt Al powder and to improve wetting ability between Al powder and SiC (or other reinforcement).

**Figure 6:**
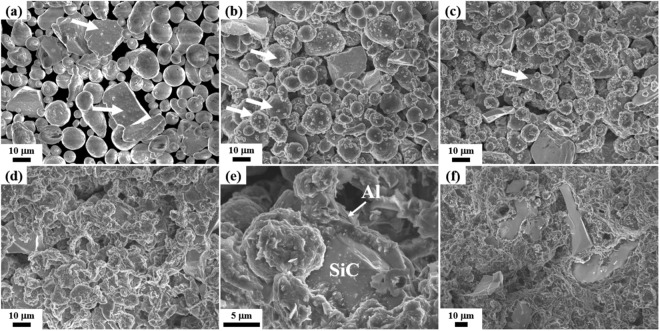
SEM images of the Al/SiC_p_ powders in a bed heated at 630 °C in nitrogen gas for **(a)** 20 min, **(b)** 30 min, **(c)** 35 min, **(d, e)** 40 min, **(f)** 60 min.

To elucidate the effect of the nitridation in detail in the course of the fabrication of AMCs, we observed changes in the degree of nitridation in the powder bed (upper figure in Fig. 5) during holding at 630 °C. The macroscopic change in the powder bed in relation to the heating time was also examined from the degree of shrinkage of the bed in the crucible after cooling. This nitridation behavior was also examined using XRD as shown in Fig. S2. When heated for 10 min, no change occurs, and the powdery state is maintained without any weight increase due to the nitridation. After heating for 20 min, the degree of nitridation is approximately 0.2%; however, the evidence of nitridation on the powder surface is not observed in this initial stage. As the heating time increases, the degree of nitridation increases from 5.2% at 40 min to 10.4% at 60 min. The bed shrinkage starts after approximately 40 min and further densification occurs with time. Figure 6 shows the scanning electron microscopy (SEM) images of the particles in the bed heated at 630 °C. With the increase in heating time, products of nitridation begin to appear on the surfaces of the Al and SiC particles, with subsequent enhancement in their amounts, as indicated by the arrows in Fig. 6. In our previous work, from the transmission emission microscopy (TEM) microstructure and electron energy loss spectroscopy (EELS) analysis of a droplet finely formed on an Al surface during nitridation, we confirmed the formation of AlN/AlON and AlN on the Al surface and inside the droplet, respectively (as shown in Fig. 1(b))^46^.

**Figure 7:**
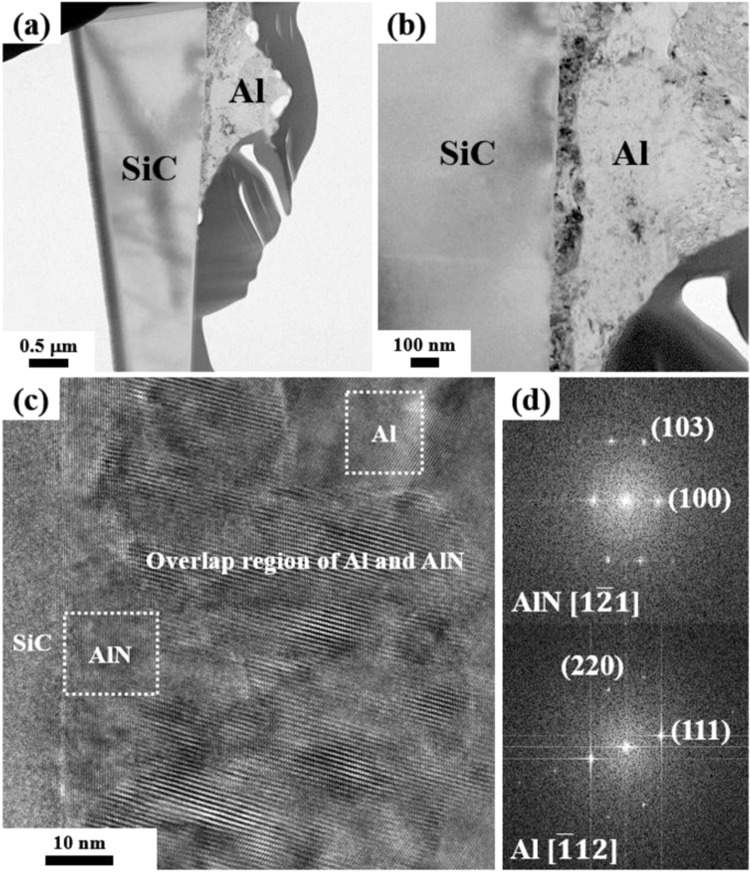
(**a, b)** Bright-field TEM images and **(c)** high-resolution TEM image of the Al/SiC_p_ in a powder bed heated at 630 °C in nitrogen gas for 40 min; **(d)** FFT patterns of the Al and AlN in (**c**).

The process of composite formation can be understood by observing the change in the shape of the Al particles as well as the degree of nitridation with time. In Fig. 6(a–c), Al maintains its particle shape, and small droplets (shown by arrows) are observed on the surface of the particles. In this state, the bed is weakly bound because sufficient liquid Al is not formed yet. As time passes, a larger amount of Al melts, and the particles no longer retain their shapes. The molten liquids become connected and induce shrinkage and densification throughout the bed (Fig. 6(d–f)). Because the heating temperature is 630 °C (which is below the melting point of Al), the melting of Al, which increases the amount of the liquid phase, can be considered to occur from the heat released by the exothermic nitridation. The liquid Al fills the pores inside the bed to cover the SiC particles, and the pores disappear, and after solidification, significant shrinkage occurs to produce dense AMCs (as shown in Fig. 1(c)). This is the most critical macroscopic composite-forming mechanism in the NISFAC process. A key feature exhibited in Fig. 6 is the excellent wetting of all the particles with liquid Al despite the well-known poor wettability between SiC and Al. As can be seen from Fig. 6(e), molten Al (marked by the arrow) maintains a very close contact with the SiC particles, indicating that the wetting is much improved by this exothermic reaction without requiring any artificial processes induced by external means. The improvement in wetting may originate from both a local temperature rise and the formation of AlN^39^. In addition, the composites produced by the NISFAC process have sufficient interfacial bond strength, and thus, show ductile fracture characteristics on the fracture surface, as shown in Fig. 6(f). We examine the cause of this in a small droplet observed in all the SEM images (Fig. 7).

A TEM sample was prepared by FIB (Focused ion beam). Figure 7 shows the TEM images of a small droplet on the SiC surface (as seen in Fig. 6(e)). As expected from Figs. 4–6, the droplets formed on the SiC particles consist of AlN and Al. AlN is formed by reacting with evaporated Al, and then grows. When the nitrogen remaining on the bed is depleted, the growth stops, and AlN is covered with the evaporated Al and becomes a droplet (Fig. 7(a,b)). The high-resolution TEM (HR-TEM) images and the corresponding fast Fourier transform (FFT) patterns (Fig. 7(c,d)) are employed to verify the phases present in the droplet. The unique structure of SiC_p_/AlN/Al will provide the best conditions in terms of wetting when liquid Al flows. Moreover, the reaction layer will have a favorable effect on the properties of the composite because it maintains a very close contact at the atomic scale. Therefore, this composite manufacturing process involves macroscopic phenomena occurring in the entire bed and microscopic phenomena occurring at the level of individual particles. First, an AlN shell is formed on the Al particle surface, as shown in Fig. 6, via the reaction with nitrogen gas. Owing to this exothermic nitridation, Al melts inside the shell. Subsequently, the shell breaks due to the thermal expansion, and liquid Al flows out^40^. The Al pool, formed from the coalescence of the flowing liquid Al, densifies while wetting the surrounding AlN shell and SiC particles. The wettability between the SiC particles and liquid Al (or the pool) is improved by the small AlN droplets formed on the SiC surfaces. Because of the improved wettability due to 1) the coarse AlN shell formation on the surface of the Al particles and 2) AlN droplet formation on the SiC surfaces, the liquid Al is trapped inside the powder and a dense composite material is formed.

## Conclusion

In summary, we develop a novel AMCs manufacturing process, termed the NISFAC process, which dramatically reduces the complex steps inevitable in numerous commercial techniques. The NISFAC process is a very simple and easy process in which the AMCs are prepared by simply mixing Al and the reinforcement powder and then heating the mixture in a nitrogen atmosphere. The wettability of the Al liquid and reinforcement, which is a precondition for the preparation of composites, is improved by the presence of small Al/AlN droplets on the reinforcement, and the Al powder surface is converted to AlN, which acts as an anchor for the Al liquid phase flow. Thus, the singular point of the NISFAC process is the simultaneous pre-existence of an Al liquid phase in the space to from a composite by using a mixed powder bed (or preform) and presence of the anchor site together with surface modification by heating in nitrogen atmosphere. Some of the key advantages of the NISFAC process are as follows. Because the nitridation of Al is exothermic, the production temperature can be lower than the melting point of Al (or the liquidus temperature, in case of an Al-alloy), thereby providing economic efficiency. In addition, because the surface modification of Al and the reinforcement phase occurs spontaneously by the nitridation, the issue of wettability between the two phases is not of concern. Hence, all types of reinforcements can be used. This opens up possibilities to tailor the characteristics of a composite by adding different types of reinforcements individually or in combination, similar to alloy design. Another advantage of the NISFAC process is the ability to freely control the distribution of the reinforcement powder (or fiber) and easily fabricate composites of various sizes and near-net shapes. In addition to the NISFAC process being simple and low-cost, the mechanical and thermal properties of the composites produced by it are comparable to of those available from conventional processes. Hence, the composites manufactured by this process can be suitably applied as structural or functional materials.

## Methods

Basically, the NISFAC process involves two simple steps, namely, i) mixing of the raw material powders and ii) heating the mixture under a nitrogen atmosphere. In this study, the AMCs were fabricated by adding various types of reinforcements (SiC, Al_2_O_3_, B_4_C, TiC, TiB_2_, and carbon fiber) to Al matrices of various compositions (pure, 2xxx, 6xxx, 7xxx Al-alloy series). The average particle size of the Al powder was 10–74 μm (Chengdu Best New Materials Co., Ltd). The average size of the reinforcement was 10–74 μm, and the volume fraction was 10–70%. Raw powders in various combinations, in terms of the size, volume fraction of reinforcement, and type of matrix, were mixed using a Turbula mixer (DM-T2, Daemyoung Enterprise Co. Ltd., Korea) without using any milling media (e.g., balls) or process control agent. Each mixed powder was placed in a graphite crucible and charged into a furnace. It was kept in nitrogen atmosphere at 630 to 700 °C for 20 to 120 min, and then taken out of the furnace and air-cooled. The heating rate was 5 °C/min. The nitrogen flow rate was maintained from 1 to 3 L/min. The gas was exhausted via a water-filled beaker to inhibit the ingress of external oxygen into the furnace. During heating of the powder mixture, nitridation occurred in the mixture bed due to the reaction between the Al powder and nitrogen atmosphere. The degree of nitridation, which has a critical impact on the characteristics of AMCs, was estimated by measuring the weight of the crucible before and after heating. The temperature change inside the mixed powder bed due to the exothermic nitridation was also measured by a data acquisition software (Lutron, SW-U801-WIN) after inserting a thermocouple in the center of the bed. The near-net-shape samples, prepared using gypsum molds, are shown in Fig. 1. The fabrication conditions for the composites shown in Figs. 1 and 2 are provided in Supplementary information.

To evaluate the mechanical properties of the synthesized AMCs, tensile tests were conducted at a strain rate of 10^−4^/s at room temperature (Instron 5967, Instron, United States) on the specimens prepared by extrusion at 350 °C (with an extrusion ratio of 18:1). The thermal expansion coefficient was measured from room temperature to 405 °C at a heating rate of 10 °C/min under a load of 30 cN (DIL 402 C, NETZCH, Germany). The thermal diffusivity (LFA457, NETZSCH, Germany) was evaluated by the laser flash method, and the density (XS205, Mettler-Toredo, Swiss) was determined by the Archimedes principle. After measuring the specific heat (DSC 200 F3, NETZSCH, Germany), the thermal conductivity was calculated using the thermal diffusivity coefficient, density, and specific heat value. The CTE was measured using a dilatometer (DIL 402 C, NETZCH, Germany) at a heating rate of 10 °C/min in the temperature range from 20–405 °C under a constant applied load of 30 N. X-ray diffraction patterns were measured by X-ray diffraction (XRD, Ultima IV, Rigaku, Japan) with a Cu K_α_ radiation source. The microstructure and interfacial structure of the synthesized AMCs were examined by optical microscopy (OM, Eclipse LV100ND, Nikon, Japan), scanning electron microscopy (SEM, JSM 2001F, JEOL, Japan), and transmission electron microscopy (TEM, Titan 80–300, FEI Co., Hillsboro, OR).

## Supplementary information

Supplementary Information.

## Data Availability

The datasets generated during and/or analyzed during the current study are available from the corresponding author on reasonable request.
