# Supplementary material for: Aluminum Matrix Composites Manufactured using Nitridation-Induced Self-Forming Process
Source: Sci Rep. 2019 Dec 31;9:20389. doi: 10.1038/s41598-019-56802-3 (PMC6938477; doi:10.1038/s41598-019-56802-3)
Supplement: Supplementary file 1 — Supplementary Information. [file 41598_2019_56802_MOESM1_ESM.pdf]

Supplementary Information

Aluminum Matrix Composites Manufactured using Nitridation-Induced Self-Forming Process

Kon-Bae Lee[1,*], Sung-Hoon Kim[2], Dae-Young Kim[1], Pil-Ryung Cha[1], Hae-Sung Kim[3], Hyun-Joo Choi[1,*], and Jae-Pyong Ahn[2,*]

[1]School of Advanced Materials Engineering, Kookmin University, 02707, Seoul, South Korea.
[2]Advanced Analysis Center, Korea Institute of Science and Technology (KIST), 02792, Seoul, South Korea.
[3]SENUS Co., Ltd., 14447, Bucheon, South Korea.
[*]Correspondence and requests for materials should be addressed to K. B. Lee (email: kblee@kookmin.ac.kr) or H. J. Choi (email: hyunjoo@kookmin.ac.kr) or J. P. Ahn (email: jpahn@kist.re.kr)

Table S1. Details on the fabrication conditions for the composites shown Fig. 1.

Sample	Matrix		Reinforcement			Process condition		
	Type	Size (μm)	Type	Size (μm)	Volume fraction (%)	Temperature (°C)	Time (min)	Flow rate of N_2 gas (L/min)
(d)-1	6061Al	30	SiC	10	17.5	650	165	3
(d)-2	6061Al	30	SiC	10	17.5	670	165	3
(d)-3	6061Al	20	SiC	10	17.5	680	120	3
(d)-4	6061Al	20	SiC	10	17.5	650	120	3
(d)-5	6063Al	70	SiC	40	45	700	120	3
(d)-6	6063Al	70	SiC	40	50	700	120	3
(e)-1	6061Al	20	SiC	20	50	670	90	3
(e)-2	6061Al	20	SiC	20	50	700	120	3
(f)	6092Al	10	B_4C	9.3	20	700	90	3
(g)	6063Al	70	SiC	14	50	660	120	3

Table S2. Details on the fabrication conditions for the composites shown Fig. 2

Sample	Matrix		Reinforcement			Process condition		
	Type	Size (μm)	Type	Size (μm)	Volume fraction (%)	Temperature (°C)	Time (min)	Flow rate of N_2 gas (L/min)
(a)	Pure	10	SiC	40	20	650	60	1
(b)	Pure	10	SiC	40	30	650	60	3
(c)	Pure	10	SiC	40	40	650	60	3
(d)	Pure	10	SiC	40	50	650	60	3
(e)	6092	74	B_4C	29.2	20	670	90	3
(f)	Pure	74	TiB_2	43	10	700	90	3
(g)	6061	10	Al_2O_3	20	20	670	90	3
(h)	6063	15	Carbon Fiber	D: 7.2, L:100	15	700	60	2

Table S3. Details on the fabrication conditions for the composites in Fig. 3

Matrix		Reinforcement		Process condition			
Type	Size (μm)	Type	Size (μm)	Volume Fraction (%)	Temperature (°C)	Time (min)	Flow rate of N2 gas (L/min)
Pure Al	10	SiC	40	20	650	60	1
Pure Al	10	SiC	40	30	650	60	3
Pure Al	10	SiC	40	40	650	60	3
Pure Al	10	SiC	40	50	650	60	3
2009Al	10	SiC	10	17.5	650	90	3
6061Al	10	SiC	10	17.5	650	90	3
6063Al	10	SiC	40	17.5	650	120	3
6063Al	10	SiC	40	25	650	120	3
6063Al	10	SiC	40	40	650	120	3
6063Al	10	SiC	40	50	650	120	3
7050Al	10	SiC	10	15	650	90	3
7075Al	10	SiC	10	17.5	650	90	3

Table S4. Experimental data for the composites in Fig. 3

**Matrix**	**Vol.%**	**YS (MPa)**	**UTS (MPa)**	**E (GPa)**	**El (%)**	**TC (W/mK)**	**CTE (μm/°C)**
Pure	20	208.6	269.3	82.7	7.2	219	16.89
Pure	30	220	300	92	1.5	175	13.74
Pure	40	208.5	215.8	92	1	200	13.17
2009	17.5	444	570	93	4.4		
6061	17.5	457	560	110	4.5		
6063	17.5	311	417	105	6.4	222	17.74
6063	25	347	440	110	4.2	208	16.28
6063	40	312	316	76	0.6	189	13.41
7075	17.5	555	653	119	2.8		
7050	15	487	606	103	4.7		

Table S5. Data for the commercial composites produced by PM process in Fig. 3

Company	Matrix	Vol.%	YS (MPa)	UTS (MPa)	E (GPa)	El (%)	TC (W/mK)	CTE (μm/°C)
DWA aluminum composites	2009	15.5	370	550	96	7		
	6092	17.5	451	514	106	7		9~17
		25	420	520	115	5		
		17.5	395	460	102	8		
	6063	50						
Aerospace Metal Composites Limited (AMC)	2124	25	480	700	115	5	150	15.5
		25	450	680	115	5	150	15.5
		25	300	470	115	5	150	15.5
		25	460	645	115	3.5	150	15.5
	6061	40	480	570	140	2.5	130	13.4
		40	440	560	140	3	130	13.4
Alyn corporation	6092	15	430	500	98	7		18.9
		25	460	540	120	4.5		15.3
		35	520	590	130	1.2		14.2
Talon Composites, Inc	6092	5	372.3	427.5	76.5	8	180	22.5
		10	386.1	441.3	84.1	6	183	21.1
		15	399.9	455.1	91.7	5	185	19.1
		20	413.7	468.9	100.7	4.5	187	17.5
		25	434.4	489.5	109.6	3	187	16.2
		30	448.2	503.3	120	1.5	189	14.9
		35	462	524	130.3	1	189	14.2
		40	468.9	537.8	142	0.8	190	13.7
	7093	5	606.8	648.1	77.9	4	131	22.5
		10	613.7	655	85.5	3	134	21.1
		15	620.6	661.9	93.8	1.5	137	19.1
Creuzet Aeronautique	2124	17	400	610	100	7		
		17	500	610	100	6		
		25	500	700	115	4		
	6013	20	450	520	104	5		
	6113	25	490	540	121	2		
	6092	25	480	520		3		
	8090	17	450	540	103	4		
Sumitomo Electric USA, Inc	Al	30		98		6.8	200	18.0
		50		147		0.5	180	12.0
		70		78		0.2	150	8.0

Table S6. Data for the commercial composites produced by stir casting in Fig. 3

**Company**	**Matrix**	**Vol. %**	**YS (MPa)**	**UTS (MPa)**	**E (GPa)**	**EL (%)**	**TC (W/mK)**	**CTE (μm/°C)**
Rio Tinto Alcan	A359	20	338	359	98.6	0.4		17.5
		10	303	338	86.2	1.2		20.7
		30	210	216	120	1		14.6
	A380	20	303	352	113.8	0.4		16.9
		10	241	345	93.8	1.2		19.3
	A360	20	248	303	108.2	0.5		16.6
		10	221	310	91	0.9		21.4
Metallic Composites for the 21st Century, Inc (MC-21)	Al	30		175				
		40		180				
		30					175	14.5
		45					180	10.5
		20			113.8		185	15.0
		30			138		180-185	13-14
		10-40			86-165		180-200	11-16

Table S7. Data for the commercial composites produced by infiltration in Fig. 3

Company	Matrix	Vol. %	YS (MPa)	UTS (MPa)	E (GPa)	EL (%)	TC (W/mK)	CTE (μm/°C)
Ametek, Inc	2014	68		210	228		220	7.5
	Al-12Si	68		225	230		226	6.8
High Performance Materials Group (HPMG)	4040	30		225	127			14.8
Advanced Refractory Technologies, Inc (ART)	A356	20	193	380	96.5			
Ceramics Process Systems (CPS)	A356	63		488	188	29.5	190	8-8.75
		37		471	167		180	10.9-11.7
	A356.2	67			217.5		180	6.9
		63			192		170	8.0
		54			167		170	9.8
Cercast	A357	65	250		160			8.5
dmc2 Electronic Components, now Ixion Thermal Materials	Al	55			235		165	7.2
					235		175	6.8
		70			225		180	6.4
					262		192	6.9
					151		183	11.9
					106		132	16.2
					196		179	9.4
					125		165	14.0
PCC Advanced Forming Technology (PCC-AFT)	Al	63		253	220			8.3
		68		207	255			7.4
		69			225			6.9
Electrovac AG	Al-7Si-0.3Mg	70			200		180-200	6~8
M - Cubed Technologies, Inc	AA360	30		317	125		150	15.6
		55		340	200		160	11.8
		70			270		170	6.2

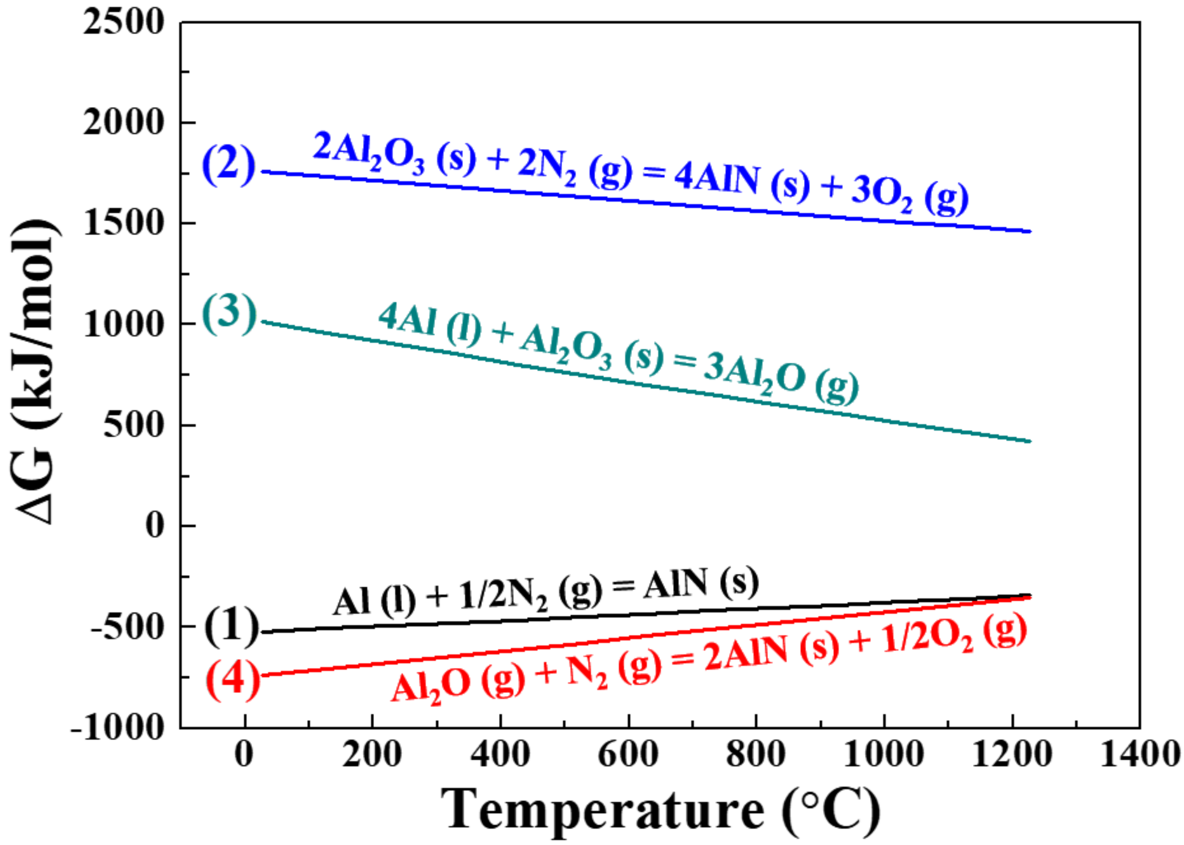

Figure S1. Thermodynamic analysis of nitridation reaction.

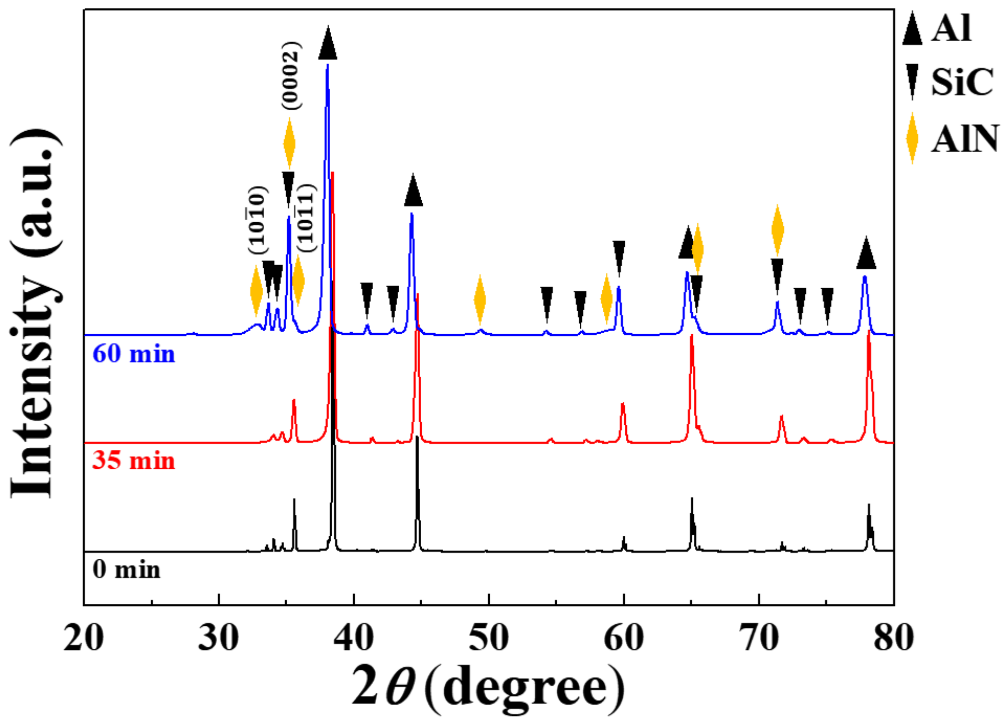

Figure S2. XRD patterns of the Al/SiC$_p$ powder beds held at 630 °C for various times.
